# Low Treatment Rates of Parasitic Diseases with Standard-of-Care Prescription Drugs in the United States, 2013–2019

**DOI:** 10.4269/ajtmh.22-0291

**Published:** 2022-08-22

**Authors:** Heesoo Joo, Brian A. Maskery, Jonathan D. Alpern, Rebecca J. Chancey, Michelle Weinberg, William M. Stauffer

**Affiliations:** ^1^ Division of Global Migration and Quarantine, Centers for Disease Control and Prevention, Atlanta, Georgia;; ^2^ HealthPartners Institute, Bloomington, Minnesota;; ^3^ Department of Medicine, University of Minnesota, Minneapolis, Minnesota;; ^4^ Division of Parasitic Diseases and Malaria, Centers for Disease Control and Prevention, Atlanta, Georgia;; ^5^ Center for Global Health and Social Responsibility, University of Minnesota, Minneapolis, Minnesota

## Abstract

To assess appropriate drug treatment of parasitic diseases in the United States, we examined the treatment rates of 11 selected parasitic infections with standard-of-care prescription drugs and compared them to the treatment rates of two more common bacterial infections (*Clostridioides difficile* and streptococcal pharyngitis). We used the 2013 to 2019 IBM^®^ MarketScan^®^ Commercial Claims and Encounters and MarketScan^®^ Multi-State Medicaid databases, which included up to 7 years of data for approximately 88 million and 17 million individuals, respectively, to estimate treatment rates of each infection. The number of patients diagnosed with each parasitic infection varied from 57 to 5,266, and from 12 to 2,018, respectively, across the two databases. Treatment rates of 10 of 11 selected parasitic infections (range, 0–56%) were significantly less than those for streptococcal pharyngitis and *Clostridioides difficile* (range, 65–85%); giardiasis treatment (64%) was comparable to *Clostridioides difficile* (65%) in patients using Medicaid. Treatment rates for patients with opisthorchiasis, clonorchiasis, and taeniasis were less than 10%. Although we could not verify that patients had active infections because of limitations inherent to claims data, including coding errors and the inability to review patients’ charts, these data suggest a need for improved treatment of parasitic infections. Further research is needed to verify the results and identify potential clinical and public health consequences.

Medical literature, mostly addressing malaria, as well as anecdotal observations from clinical experience suggest that the diagnosis and treatment of parasitic diseases in countries where these infections are not endemic, including the United States, are frequently missed, delayed, or inadequate.^1^^–^^4^ The inappropriate, missed, or delayed diagnosis and treatment of parasitic infections in nonendemic countries could be the result of multiple factors, such as failure to obtain travel or migration history, healthcare providers’ lack of knowledge about specific pathogens and where they are endemic, providers’ lack of familiarity with diagnostic tests available and/or how to interpret test results, and expense or lack of availability of necessary treatments.^5^^,^^6^ Furthermore, the U.S. populations that suffer from these infections disproportionately often have additional barriers to obtaining health care, such as lower rates of insurance coverage (more out-of-pocket expenses), physical barriers (e.g., limited transportation), cultural and language barriers, and lack of trust in medical providers and systems.^7^^–^^9^ The dramatically increasing cost of many antiparasitic drugs used to treat these infections has become an additional barrier in recent years.^10^

Low treatment rates among patients diagnosed with soil-transmitted helminth infections (ascariasis, trichuriasis, and hookworm) have been reported previously in the United States.^11^ Our study examined the treatment rates of a broad range of parasitic infections compared with treatment rates of other more common infections in the United States. We also assessed the treatment of specific infections by type of insurance (private versus Medicaid).

We investigated the treatment of 11 parasitic diseases, including opisthorchiasis, clonorchiasis, paragonimiasis, taeniasis, schistosomiasis, three soil-transmitted helminthiases (hookworm, trichuriasis, and ascariasis), amebiasis, strongyloidiasis, and giardiasis. We selected two common comparator infections: *Clostridioides difficile* and streptococcal pharyngitis. Our selection of both the parasitic infections and the controls was based on similarities in having definitive outpatient diagnoses, having definitive outpatient treatment courses with a limited number of outpatient/oral drugs available, and a defined and short treatment course.

We included data from patients with a first diagnosis of each infection in the IBM^®^ MarketScan^®^ Commercial Claims and Encounters Database and in the MarketScan^®^ Multi-State Medicaid Database (IBM^®^ Watson Health, Ann Arbor, MI) between January 1, 2013 and December 31, 2019 using the online tool, Truven Health MarketScan Treatment Pathways. For 2013 through 2019, the MarketScan^®^ Commercial Claims and Encounters Database includes approximately 88 million individuals with employer-sponsored private health insurance; the MarketScan^®^ Multi-State Medicaid Database includes 17 million Medicaid-covered individuals. Note that these individuals may not have been included in the databases during the entire study period.

We defined each infection using the International Classification of Diseases, Ninth Revision, Clinical Modification (ICD-9-CM) codes between January 1, 2013 and September 30, 2015, and ICD-10-CM codes between October 1, 2015 and December 31, 2019 (Table 1). We excluded rule-out diagnoses (i.e., claims with the selected ICD diagnosis codes that the database administrators determined to be for laboratory tests only). For each infection, we defined standard-of-care (SOC) prescription drugs based on expert opinion or clinical trials when available (Table 1).^12^^–^^18^

**Table 1:** International Classification of Diseases, Ninth Revision, Clinical Modification (ICD-9-CM) and ICD-10-CM codes, and standard-of-care (SOC) prescription drugs by infection

Infection	ICD-9-CM codes	ICD-10-CM codes	SOC prescription drugs
Opisthorchiasis	121.0	B66.0	Praziquantel, albendazole*
Clonorchiasis	121.1	B66.1	Praziquantel, albendazole*
Paragonimiasis	121.2	B66.4	Praziquantel, triclabendazole†
Taeniasis	123.0, 123.2, 123.3	B68, B68.0, B68.1, B68.9	Praziquantel, niclosamide, albendazole‡
Schistosomiasis	120.0, 120.1, 120.2, 120.8, 120.9	B65, B65.0, B65.1, B65.2, B65.8, B65.9	Praziquantel
Hookworm	126.0, 126.1, 126.2, 126.3, 126.8, 126.9	B76, B76.0, B76.1, B76.8, B76.9	Albendazole, mebendazole§
Amebiasis	006, 006.0, 006.1, 006.2, 006.3, 006.4, 006.5, 006.6, 006.8, 006.9	A06, A06.0, A06.1, A06.2, A06.3, A06.4, A06.5, A06.6, A06.7, A06.8, A06.81, A06.82, A06.89, A06.9	Metronidazole, tinidazole, paromomycin sulfate, iodoquinol
Trichuriasis	127.3	B79	Albendazole, mebendazole, ivermectin^‖^
Strongyloidiasis	127.2	B78, B78.0, B78.1, B78.7, B78.9	Ivermectin, albendazole¶
Ascariasis	127.0	B77, B77.0, B77.8, B77.81, B77.89, B77.9	Albendazole, mebendazole, ivermectin#
Giardiasis	007.1	A07.1	Metronidazole, tinidazole, nitazoxanide,
Clostridioides difficile	008.45	A047, A0472	Vancomycin hydrochloride, fidaxomicin, metronidazole
Streptococcal pharyngitis	034.01	J02.0	Penicillin V, amoxicillin, cephalexin, cefadroxil, clindamycin, azithromycin, clarithromycin

* Albendazole is an alternative drug for the treatment of opisthorchiasis or clonorchiasis infections, although is not U.S. Food and Drug Administration (FDA)-approved for this purpose (off-label use).

† Triclabendazole is an alternative drug.

‡ Niclosamide is an alternative drug for treatment of taeniasis, but it is not available for human use in the United States. Albendazole is another option based on studies treating a small number of infected individuals. Neither praziquantel nor albendazole are FDA-approved for treating taeniasis (off-label use).

§ Pyrantel pamoate, an over-the-counter drug in the United States, is also recommended for treatment of hookworm. Albendazole is effective in treating hookworm but is not FDA-approved for this purpose.

‖ Albendazole and ivermectin are effective in treating trichuriasis; however, neither is FDA-approved for this purpose. The safety of ivermectin for children weighing less than 15 kg has not been established.

¶ Albendazole is an alternative drug for treating acute and chronic strongyloidiasis.

# Albendazole is effective in treating ascariasis, but is not FDA-approved for this purpose. The safety of ivermectin for children weighing less than 15 kg has not been established.

For each infection, we included patients covered by private insurance or Medicaid only. We excluded patients potentially covered by Medicare, including those enrolled in employer-sponsored Medicare supplemental plans (private insurance sample) or those 65 years or older at the first diagnosis (Medicaid sample) (Figure 1). We excluded patients if they were not enrolled continuously in private insurance or Medicaid from 30 days before and for 90 days after the first diagnosis. We excluded hospitalized patients and those with multiple parasitic infections because of the complexity of determining the SOC. We estimated treatment rates as follows: we classified patients as treated with SOC prescription drugs if they had insurance drug claims for at least one indicated SOC prescription drug (Table 1) between 30 days before and up to 90 days after their first diagnosis. We did not consider treatment with non-SOC prescription drugs. We defined the treatment rate for each infection as the number of patients treated with at least one indicated SOC drug divided by the total number of patients diagnosed for a given infection. For each infection, we compared treatment rates among those who had private insurance with treatment rates for those with Medicaid by using odds ratios. We also compared treatment rates for the 10 parasitic infections and treatment rates for two common infections after subdividing by insurance type (private insurance and Medicaid) using odds ratios. Odds ratios for opisthorchiasis were not estimated because of zero count cells.

**Figure 1.**
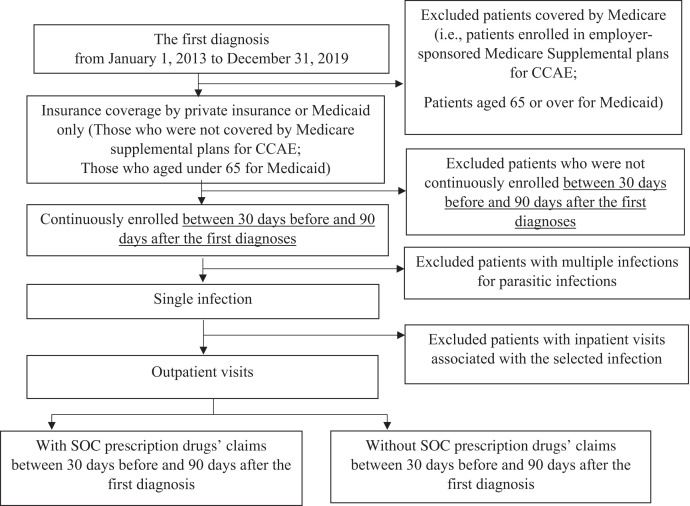
Selection of data based on procedure codes the for MarketScan^®^ Commercial Claims and Encounters (CCAE) Database and the MarketScan^®^ Multi-State Medicaid Database using the Truven Health MarketScan Treatment Pathways, 2013 to 2019. SOC = standard-of-care.

Sample sizes for each of 11 parasitic diseases were relatively small, ranging from 12 (Medicaid patients with a diagnosis of opisthorchiasis) to 5,266 (privately insured patients with a diagnosis of *Giardia*). Sample sizes for *C. difficile* were 76,893 for private insurance and 13,705 for Medicaid. Sample sizes for streptococcal pharyngitis were approximately 3.2 million for private insurance and 1.8 million for Medicaid. Treatment rates were generally low and varied widely among the 11 parasitic diseases, ranging from 0% to 70%. Of note, treatment rates for patients with opisthorchiasis, clonorchiasis, and taeniasis diagnoses were less than 10% regardless of whether patients used private insurance or Medicaid (Table 2). Among parasitic infections, the greatest treatment rates (70% with private insurance and 64% with Medicaid) were observed among patients with giardiasis diagnoses.

**Table 2:** Comparison of treatment rates with standard-of-care prescription drugs by insurance type, 2013 to 2019

Infection	Private insurance		Medicaid		Odds ratio (P/M)	95% CI
	*n*	Treatment rate, %	*n*	Treatment rate, %		
Opisthorchiasis	94	0	12	0	NA	NA
Clonorchiasis	76	3	58	3	0.76	0.05–10.76
Paragonimiasis	57	4	19	32	0.08	0.01–0.53
Taeniasis	1,229	6	614	5	1.20	0.77–1.90
Schistosomiasis	573	19	245	31	0.53	0.37–0.75
Hookworm	2,569	29	1,153	23	1.36	1.16–1.61
Amebiasis	2,967	37	761	25	1.74	1.45–2.10
Trichuriasis	174	41	142	52	0.63	0.40–1.02
Strongyloidiasis	702	41	397	43	0.92	0.72–1.20
Ascariasis	1,476	56	1,341	52	1.17	1.00–1.36
Giardiasis	5,266	70	2,018	64	1.31	1.17–1.46
*Clostridioides difficile*	76,893	73	13,705	65	1.46	1.40–1.51
Streptococcal pharyngitis	3,224,539	81	1,841,312	85	0.76	0.76–0.77

M = Medicaid; NA = not available because of zero cell counts; P = private insurance. Odds ratios > 1.0 indicate greater treatment rates with private insurance vs. Medicaid; odds ratios < 1.0 indicate greater treatment rates with Medicaid.

Treatment rates varied by insurance type; however, they were not consistently greater for one type of insurance. Compared with patients using Medicaid insurance, those with private insurance were more likely to be treated with SOC prescription drugs when diagnosed with hookworm (odds ratio [OR], 1.36; 95% confidence interval [CI], 1.16–1.61), amebiasis (OR, 1.74; 95% CI, 1.45–2.10), ascariasis (OR, 1.17; 95% CI, 1.00–1.36), giardiasis (OR, 1.31; 95% CI, 1.17–1.46), and *C. difficile* (OR, 1.46; 95% CI, 1.40–1.51) (Table 2). In contrast, compared with patients using Medicaid, those with private insurance were less likely to be treated with prescription SOC drugs when diagnosed with paragonimiasis (OR, 0.08; 95% CI, 0.01–0.53), schistosomiasis (OR, 0.53; 95% CI, 0.37–0.75), and streptococcal pharyngitis (OR, 0.76; 95% CI, 0.76–0.77).

The treatment rates for the two common infections were much greater than for the parasitic infections: 65% to 73% for *C. difficile* and 81% to 85% for streptococcal pharyngitis. For 10 parasitic infections, treatment rates with SOC prescription drugs after diagnoses were less (often much less) than for patients with diagnoses of *C. difficile* or streptococcal pharyngitis. Patients on Medicaid with a diagnosis of giardiasis had similar treatment rates compared with patients on Medicaid with a diagnosis of *C. difficile* (Tables 2 and 3).

**Table 3:** Comparison of treatment rates with standard-of-care prescription drugs for 10 parasitic infections compared with each of two common infections, *Clostridioides difficile* and streptococcal pharyngitis, subdivided by insurance type, 2013 to 2019

Parasitic infection	*Clostridioides difficile*		Streptococcal pharyngitis	
	Private insurance, odds ratio, P/C (95% CI)	Medicaid, odds ratio, P/C (95% CI)	Private insurance, odds ratio, P/S (95% CI)	Medicaid, odds ratio, P/S (95% CI)
Clonorchiasis	0.01 (0.001–0.04)	0.02 (0.002–0.07)	0.01 (0.001–0.02)	0.01 (0.001–0.02)
Paragonimiasis	0.01 (0.002–0.05)	0.25 (0.08–0.71)	0.01 (0.001–0.03)	0.08 (0.03–0.24)
Taeniasis	0.02 (0.02–0.03)	0.03 (0.02–0.04)	0.02 (0.01–0.02)	0.01 (0.01–0.01)
Schistosomiasis	0.09 (0.07–0.11)	0.24 (0.18–0.32)	0.06 (0.04–0.07)	0.08 (0.06–0.11)
Hookworm	0.15 (0.14–0.17)	0.16 (0.14–0.19)	0.10 (0.09–0.11)	0.05 (0.05–0.06)
Amebiasis	0.22 (0.20–0.24)	0.18 (0.15–0.22)	0.14 (0.13–0.15)	0.06 (0.05–0.07)
Trichuriasis	0.26 (0.19–0.35)	0.60 (0.42–0.84)	0.17 (0.12–0.23)	0.20 (0.14–0.28)
Strongyloidiasis	0.26 (0.23–0.31)	0.41 (0.34–0.51)	0.17 (0.14–0.20)	0.14 (0.11–0.17)
Ascariasis	0.47 (0.42–0.52)	0.59 (0.52–0.66)	0.30 (0.27–0.33)	0.20 (0.18–0.22)
Giardiasis	0.90 (0.84–0.95)	0.99 (0.90–1.10)	0.57 (0.54–0.61)	0.33 (0.30–0.36)

C = *Clostridioides difficile*; P = parasitic infection; S = streptococcal pharyngitis. Odds ratios for opisthorchiasis were not estimated because of zero count cells.

The treatment rates for most parasitic infections examined in our study were low compared with the more common diagnoses of *C. difficile* or streptococcal pharyngitis and varied by insurance type. For three infections (paragonimiasis, schistosomiasis, and trichuriasis), treatment rates were much greater among Medicaid patients; for amebiasis, treatment rates were much greater among privately insured patients. Among many potential reasons for low treatment rates among patients with parasitic infections is the dramatic increase in the prices of many antiparasitic drugs, including praziquantel, albendazole, and mebendazole.^11^ A previous study^11^ found that the treatment rates of ascariasis and trichuriasis decreased as drug prices increased from 2010 to 2017, and that the quality of care for hookworm decreased as substitution from SOC drugs to non-SOC drugs was observed. In addition, several parasitic infections have limited or no alternative drug treatments. In our study, we were limited to identifying filled prescriptions using only drug claims data; the actual number of prescriptions written is unknown. Thus, one of the possible contributing factors to low treatment rates among patients with parasitic infections might be low rates of filled prescriptions, which could be related to the high cost of medications.

Limitations of this study include missing data or coding errors inherent in the use of claims data.^19^ Misdiagnoses and coding errors—including mistyping treatment or diagnosis codes, or recording a previously treated infection from a patient’s chart—may be proportionately greater for these less familiar and uncommon infections with small numbers of diagnoses. Such errors may contribute to the underestimation of treatment rates. Payments for over-the-counter drugs, which may be recommended for parasitic diseases, are not available in the MarketScan^®^ datasets. For instance, pyrantel pamoate is available over-the-counter and is recommended for treating hookworm in the United States. Thus, the reported treatment rates for hookworm may be underestimated.^20^ In addition, because of costs, clinicians may be assisting or recommending patients receive medications by ordering through the Internet or mail, or by importing by family or friends. We considered patients as treated with prescription SOC drugs if there was documentation of any single SOC medication, but some infections require multiple medications for adequate treatment, which could lead to our overestimating some treatment rates. For example, amebic liver abscesses require metronidazole followed by an intraluminal agent. Although the MarketScan^®^ databases included a large portion of the U.S. population, the data are not nationally representative. For instance, the database does not include uninsured individuals, who may face additional barriers and have lower treatment rates than those with health insurance. Also, a limited number of states, 5 to 10 states during the study period, provided data to MarketScan^®^ Multi-State Medicaid database, and the states included in the datasets have changed over time. The composition of states included may affect overall estimates. Last, we could not examine the causality of the low use of SOC prescription drugs.

Despite these limitations, the strikingly low SOC treatment rates of selected parasitic infections are concerning. There are limited data on reasons for treatment delays or missed or inappropriate treatment of parasitic diseases in the United States. These results suggest a need to investigate further the treatment rates and health outcomes for parasitic infections.
